# Severe Calorie Restriction Reduces Cardiometabolic Risk Factors and Protects Rat Hearts from Ischemia/Reperfusion Injury

**DOI:** 10.3389/fphys.2016.00106

**Published:** 2016-04-08

**Authors:** Dirceu S. Melo, Liliane V. Costa-Pereira, Carina S. Santos, Bruno F. Mendes, Karine B. Costa, Cynthia Fernandes F. Santos, Etel Rocha-Vieira, Flávio C. Magalhães, Elizabethe A. Esteves, Anderson J. Ferreira, Sílvia Guatimosim, Marco F. Dias-Peixoto

**Affiliations:** ^1^Programa Multicêntrico de Pós Graduação em Ciências Fisiológicas, Sociedade Brasileira de FisiologiaSão Paulo, Brasil; ^2^Faculdade de Medicina, Campus JK, Universidade Federal dos Vales do Jequitinhonha e MucuriDiamantina, Brasil; ^3^Faculdade de Ciências Biológicas e da Saúde, Universidade Federal dos Vales do Jequitinhonha e MucuriDiamantina, Brasil; ^4^Departamento de Morfologia, Universidade Federal de Minas GeraisBelo Horizonte, Brasil; ^5^Departamento de Fisiologia e Biofísica, Universidade Federal de Minas GeraisBelo Horizonte, Brasil

**Keywords:** cardiac function, cardiometabolic risk factors, cardioprotection, severe calorie restriction, oxidative stress

## Abstract

**Background and Aims:** Recent studies have proposed that if a severe caloric restriction (SCR) is initiated at the earliest period of postnatal life, it can lead to beneficial cardiac adaptations later on. We investigated the effects of SCR in Wistar rats from birth to adult age on risk factors for cardiac diseases (CD), as well as cardiac function, redox status, and HSP72 content in response to ischemia/reperfusion (I/R) injury.

**Methods and Results:** From birth to the age of 3 months, CR50 rats were fed 50% of the food that the *ad libitum* group (AL) was fed. Food intake was assessed daily and body weight were assessed weekly. In the last week of the SCR protocol, systolic blood pressure and heart rate were measured and the double product index was calculated. Also, oral glucose and intraperitoneal insulin tolerance tests were performed. Thereafter, rats were decapitated, visceral fat was weighed, and blood and hearts were harvested for biochemical, functional, tissue redox status, and western blot analyzes. Compared to AL, CR50 rats had reduced the main risk factors for CD. Moreover, the FR50 rats showed increased cardiac function both at baseline conditions (45% > AL rats) and during the post-ischemic period (60% > AL rats) which may be explained by a decreased cardiac oxidative stress and increased HSP72 content.

**Conclusion:** SCR from birth to adult age reduced risk factors for CD, increased basal cardiac function and protected hearts from the I/R, possibly by a mechanism involving ROS.

## Introduction

Cardiac diseases (CD) are the main leading cause of mortality worldwide accounting for up to 30% of all deaths (Go et al., 2014; Nichols et al., 2014). High-calorie plays a significant role in the development of CD by raising risk factors, such as dyslipidemia, high blood pressure, and hyperinsulinemia (Bornfeldt and Tabas, 2011). A great body of evidence has shown that limiting caloric intake is an effective non-pharmacological strategy to reduce cardiometabolic risk factors and to improve heart function delaying the onset of CD.

The cardioprotective effects of calorie restriction (CR) are well-documented for a long-term moderate CR (20–40% reduction) (Han et al., 2012; Peart et al., 2012; Noyan et al., 2015). However, few studies have focused on the effects of a long-term severe CR (SCR) (≥ 40%) on the heart, and the results are controversial. Some groups have reported positive effects of SCR on cardiac function under basal conditions (Han and Ren, 2010; Melo et al., 2013) and after cardiac insult (Yamagishi et al., 2010). However, other studies have found no significant effects (Long et al., 2002), or even undesirable effects (Okoshi et al., 2001; Sugizaki et al., 2009).

A recent meta-analysis conducted by Han and Ren (2010), showed that the effects of CR are strongly associated with age, being positive when CR is started at earliest ages. In this context, the use of a long-term SCR initiated since the postnatal period would lead to a greater reduction in the metabolism later in life, forcing the heart to increase its contractile efficiency due to a reduced energy availability.

In our previous study, we demonstrated that young adult rats (90-day old) submitted to a 50% CR since the first weeks of age presented increased basal heart function, increased cardiomyocyte density and number as well as increased levels of phosphorylated AKT (Melo et al., 2013). Based on this knowledge, we hypothesized that SCR started at the earliest period of the rodents' postnatal life and maintained until adulthood might lead to beneficial effects. The purpose of this study was to investigate: (i) if SCR rats present improvement on cardiometabolic risk factors and (ii) if SCR protects hearts against ischemia-reperfusion (I/R). Additionally, we evaluated the cardiac redox status and heat shock protein 72 (HSP72) expression as possible protective mechanisms in post-ischemic SCR-hearts.

## Methods

Experimental protocols were performed in accordance with the Guide for the Care and Use of Laboratory Animals published by the US National Institutes of Health (NIH Publication, 1996). The experimental protocols were approved by Ethics Committee on Animal Use/Federal University of Jequitinhonha and Mucuri Valleys, Diamantina, MG, Brazil, protocol #04/2015.

### Animals and caloric restriction protocol

Female pregnant Wistar rats (*n* = 8), approximately 90 days of age, were housed in individual cages and maintained in a room with controlled temperature (22 ± 2°C) and a 12 h light–12 h dark cycle, with free access to food and water. After they gave birth, half of the mothers (*n* = 4) were assigned to *ad libitum*-fed group, and the other half (*n* = 4) was assigned to the calorie-restricted group. Both groups had free access to water. In the mothers assigned to the calorie-restricted group, the amount of food provided on a daily basis was equivalent to 50% of the amount consumed by the mothers in the *ad libitum*-fed group, and each mother suckled 8 newborn rats for 21 days. After weaning, rats were housed in individual cages and received the same treatment as their mothers up to the age of 90 days. The weaned rats were divided into rats fed *ad libitum* (AL rats; *n* = 9) and rats subjected to long-term SCR (CR50 rats; *n* = 11).

### Food intake, weight gain, and feed efficiency ratio

The food intake of the AL group was evaluated daily and this value was used to calculate the offer to the CR50 group. Body weight was monitored weekly. Feed efficiency ratio was measured from the ratio between the total weight gain and overall food intake.

### Systolic blood pressure, heart rate, and double product ratio

The rats were previously familiarized with the procedures for tail-cuff plethysmography for 5 days (MLT1020PPG IR Plethysmograph, ADInstruments). Tail systolic blood pressure and heart rate were measured at the last week prior to the end of the experiment. Additionally, we calculated the double product index using systolic blood pressure and heart rate values, an indicative of cardiac work (Schutte et al., 2013).

### Basal metabolic rate

After an overnight fast (12 h), the resting metabolic rate was assessed by the average oxygen consumption (VO_2_) and carbon dioxide production (VCO_2_) within a period of 60 min. The measurements were taken by a computer-monitored indirect calorimeter (Oxyleptro, Harvard Apparatus, Spain) coupled to a metabolic chamber (air flow = 1.0/min), which accommodated the rats. The calorimetric parameters were measured using a respiratory-based software program (software Metaoxy, Harvard Apparatus, Spain).

### Oral glucose tolerance test (OGTT)

Immediately after the basal metabolic rate measurement, glucose was administered by gavage (2 g/kg body weight; 50% solution). Blood glucose levels were determined by small clipping of the rat tail, immediately before (0 min) the glucose challenge, as well as at 30, 60, and 120 min thereafter. Blood glucose levels were determined using an ACCU-CHEK (Advantage Glucose Analyzer, Roche Diagnostics Corporation, IN, USA).

### Intraperitoneal insulin tolerance test (IpITT)

Forty-eight hours after the *OGTT*, the animals were fasted for 8 h, then an intraperitoneal injection of insulin (1 IU/kg body weight) was administered. Blood glucose levels were determined following the same *OGTT* protocol.

### *Ex vivo* analysis and langendorff preparation

The animals were anesthesized with ketamine (10 mg/kg) and xylazine (5 mg/kg) and decapitated 10–15 min after an intraperitoneal injection of 400 IU heparin. The hearts were perfused in a Langendorff apparatus (ML785B2, ADInstruments) and left ventricular pressure (± dP/dt) was continuously recorded according to the Langendorff technique. Briefly, the thorax was opened and the heart was carefully dissected and rapidly cannulated and retrogradely perfused with Krebs–Ringer solution (in mmol·L^−1^: NaCl, 118.40; KCl, 4.70; KH2PO4, 1.17; MgSO4, 1.17; CaCl2, 2.50; glucose, 11.65; and NaHCO3, 26.30) at 37 ± 1°C, with constant pressure (65 mm Hg) and oxygenation (5% CO2 and 95% O2). A water-filled latex balloon coupled to a pressure transducer (MLT0380, ADInstruments) was inserted into the left ventricular cavity via the left auricle to record pressure. The rate of pressure development (± dP/dt) was obtained by calculating derivatives from the pressure variation recordings, using the Labchart8 software. After 20 min of stabilization in the perfusion system, the hearts were evaluated at baseline cardiac function for 20 min and then subjected to global ischemia (total blockage of the Krebs Ringer solution flow) for 20 min. After induction of ischemia, the hearts were reperfused for an additional 20 min to evaluate the post-ischemic cardiac function. All the ± dP/dt measurements were normalized to heart weight.

### Organ weights and adiposity index

Liver, kidney, spleen, epididymal, and retroperitoneal fat was removed and weighted. The adiposity index was calculated as following: [epididymal fat + retroperitoneal fat/(body weight–sum of fat pads)] ^*^ 100 as described by Boustany et al. (2004).

### Blood lipids analyses

Blood was centrifuged to obtain plasma, and then aliquoted in eppendorf tubes and kept at −80°C until analyses. Total plasma cholesterol (CHOL), high-density lipoprotein cholesterol (HDL-C) and triglyceride (TG) levels were determined using commercial kits (LabTest®), according to the specifications of the manufacturer and using a semi-automatic biochemical analyzer (PW-3000, PIOWAY, China). The low-density lipoprotein cholesterol (LDL-C) level was also estimated by using the Friedewald equation (Friedewald et al., 1972) and very low density lipoprotein (VLDL-C) was calculated from the triglyceride values and atherogenic index as described by Nwagha et al. (2010).

### Cardiac redox status

Left cardiac ventricle samples were weighed, immersed in phosphate-buffered saline (PBS), pH 7.2, extensively washed to remove blood and stored at −80°C until the time of assay. At the time of assay, samples were then homogenized (T 20 basic ULTRA-TURRAX; IKA Labortechnik, China) for 3 min at 0–4°C. After this, the homogenate was centrifuged for 10 min at 10,000 xg (Jouan BR4i, Thermo Fischer Scientific, USA), and the supernatant was used for the biochemical assays described below. Protein concentration for all assays was determined according to Bradford (1976) using bovine serum albumin (BSA, Sigma, USA) as standard.

The assay to determine SOD activity (EC 1.15.1.1) was performed according to Marklund and Marklund (1974) with some modifications. In brief, sample of tissue homogenate was added in 50 mM sodium phosphate buffer (1 mL, pH 7.8, 37°C) containing 1 mM diethylenetriamine pentaacetic acid (DTPA, Sigma, USA). The reaction was started by adding 0.2 mM pyrogallol (Sigma, USA), and the samples were heated to 37°C for 3 min. The absorbance was determined at 420 nm (Shimadzu UV 2450, Shimadzu Corporation, Japan). SOD activity was calculated as units per milligram of protein, and one unit of enzyme was considered as being the amount that caused inhibition of pyrogallol autoxidation by 50%. Measurements were performed in triplicate.

Catalase (EC 1.11.1.6) activity was measured in the supernatants as described by Nelson and Kiesow (1972). Briefly, 0.04 ml of H2O2 was added as substrate to 0.06 ml of tissue homogenate and 2 ml of potassium phosphate buffer (50 mM, pH 7.0) to give a final H2O2 concentration of 6 mM, and the reaction proceeded for 1 min at room temperature. Decomposition of H2O2 by catalase was noted by the change in absorbance at 240 nm (DE) (Shimadzu UV 2450, Shimadzu Corporation, Japan). Experiments were performed in triplicate. Catalase activity was expressed by millimoles of H2O2 decomposed per minute per milligram of protein (E × min^−1^× mg^−1^ protein). This procedure avoids the possible interference associated with glutathione peroxidase activity since the necessary cofactors are not present in the reaction media.

As an index of lipid peroxidation, we used the formation of thiobarbituric acid-reactive substance (TBARS) during an acid-heating reaction (Ohkawa et al., 1979). Briefly, 0.4 ml of tissue homogenate was added to 0.2 ml of 8.1% sodium dodecyl sulfate, 0.5 ml of acetic acid (2.5 M, pH 3.4), and 1 ml of 0.8% thiobarbituric acid (Sigma, USA), and they were then heated in a boiling water bath (90°C) for 60 min. Samples then were centrifuged for 5 min at 50,000 g (Jouan BR4i, Thermo Fischer Scientific, USA). TBARS were determined by the absorbance at 532 nm (Shimadzu UV 2450, Shimadzu Corporation, Japan), compared with a standard curve constructed with known concentrations of malondialdehyde (1,1,3,3-tetramethoxy- propane) (Sigma, USA) as an external standard. The amount of malondialdehyde (MDA) produced was interpreted as the TBARS levels and indicates the degree of oxidative stress. The results are expressed as MDA equivalents per milligram of protein. Measurements were performed in triplicates.

### Western blot

Total protein content of left cardiac ventricles was quantified with the Bradford protein assay (Bradford, 1976). Protein (50 μg) was loaded onto a 10% polyacrylamide gel for electrophoresis. After electrophoresis, proteins were transferred to a PVDF membrane, blocked with a 5% nonfat milk solution, and washed in phosphate-buffered saline, containing 0.1% Tween 20. Membranes were incubated overnight at 4°C with the following primary antibodies: Hsp72 (1:5000 dilution–Enzo Life Sciences) and glyceraldehyde 3-phosphate dehydrogenase (GAPDH, 1:5000 dilution–Cell Signaling) Thereafter, a monoclonal anti-rabbit or anti-mouse secondary antibody conjugated with peroxidase (1:7500 dilution, Cell Signaling) were used. Immunodetection was carried out using enhanced chemiluminescence (Amersham Biosciences), and protein levels were expressed as a ratio of optical densities.

### Statistical analysis

All data are expressed as means ± standard deviation. Statistical significance was estimated using Student's *t* test or ANOVA, followed by Tukey's *post-hoc* test (GraphPad Prism 4.0). The level of significance was set at *p* < 0.05.

## Results

The characteristics of the groups are presented in Table 1. As expected, CR50 rats had a 40% lower final body weight compared to AL rats. Additionally, feed efficiency ratio was 18% higher in CR50 rats, which may be explained by a reduced basal oxygen consumption of these animals. Also, most organ weights were reduced in CR50 rats. Interestingly, although heart weight of CR50 was lower, we found similar heart/body weight ratio in these rats when compared to AL rats.

**Table 1:** **General characteristics of *Ad Libitum* (AL) and 50% SCR (CR50) rats**.

	**AL**	**CR50**
	**(*n* = 9)**	**(*n* = 11)**
Final body weight (g)	288.2 ± 10.0	167.1 ± 3.5^*^
Final food intake (g)	1489 ± 38	746 ± 19^*^
Feed efficiency ratio (%)	19.4 ± 0.6	23.0 ± 0.2^*^
VO2 (ml/min/Kg^∧0.75^)	22.5 ± 0.5	20.5 ± 0.7^*^
Heart (g)	1.2 ± 0,1	0.7 ± 0.1^*^
Heart/Body (mg/g)	4.4 ± 0.2	4.2 ± 0.1
Liver (g)	10.7 ± 1.7	5.9 ± 0.4 ^*^
Kidneys (g)	1.1 ± 0.1	0.6 ± 0.1 ^*^
Spleen (g)	0.8 ± 0.1	0.5 ± 0.1 ^*^

*Data are presented as mean ± SD*.

* *P < 0.05. AL vs. CR50. T student test*.

Next, we evaluated the effects of 50% CR on key risk factors for developing CD (Table 2). We found that most of these cardiometabolic risk factors were reduced in CR50 rats. Specifically, when compared to AL, CR50 rats had reduction in adiposity index and visceral fat weight, improvement in plasma lipid profile, as shown by increased HDL cholesterol, decreased LDL and atherogenic index. Also, CR50 rats had an improvement in glucose tolerance and insulin response, which indicates higher insulin sensitivity. Cardiovascular parameters also indicate protective effects of CR. CR50 rats had lower systolic blood pressure and cardiac double product index compared to AL rats.

**Table 2:** **Severe calorie restriction reduces cardiometabolic risk factors. ***Ad Libitum*** (AL) and 50% SCR (CR50) rats**.

	**AL**	**CR50**
	**(*n* = 9)**	**(*n* = 11)**
Visceral fat (g)	11.3 ± 1.9	2.5 ± 0.2^*^
Adiposity index (%)	3.9 ± 0.3	1.5 ± 0.1^*^
Triglycerides (mg/dL)	111.1 ± 5.6	106.4 ± 4.2
Cholesterol (mg/dL)	61.5 ± 3.1	61.4 ± 2.7
HDL—cholesterol (mg/dL)	20.2 ± 0.9	23.4 ± 0.8^*^
LDL—cholesterol (mg/dL)	24.0 ± 4.0	14.3 ± 0.8^*^
VLDL—cholesterol (mg/dL)	23.8 ± 1.8	21.2 ± 0.8
Atherogenic index (mg/dL)	3.0 ± 0.1	2.4 ± 0.1^*^
Basal glucose (mg/dL)	97.8 ± 3.0	94.3 ± 1.0
Area under curve OGTT (mg/dL/min)	14410 ± 311	13280 ± 265^*^
Area under curve ITT (mg/dL/min)	269.9 ± 10.3	208.7 ± 9.4^*^
Systolic blood pressure (mmHg)	134.9 ± 3.8	123.2 ± 2.6^*^
Heart rate *in vivo* (bpm)	402 ± 11	382 ± 7
Double product index (mmHg^*^bpm)	55490 ± 1516	46640 ± 1655^*^

* Data are presented as mean ± SD. ^*^ P < 0.05 AL vs. CR50. T student test.

**Figure 1:**
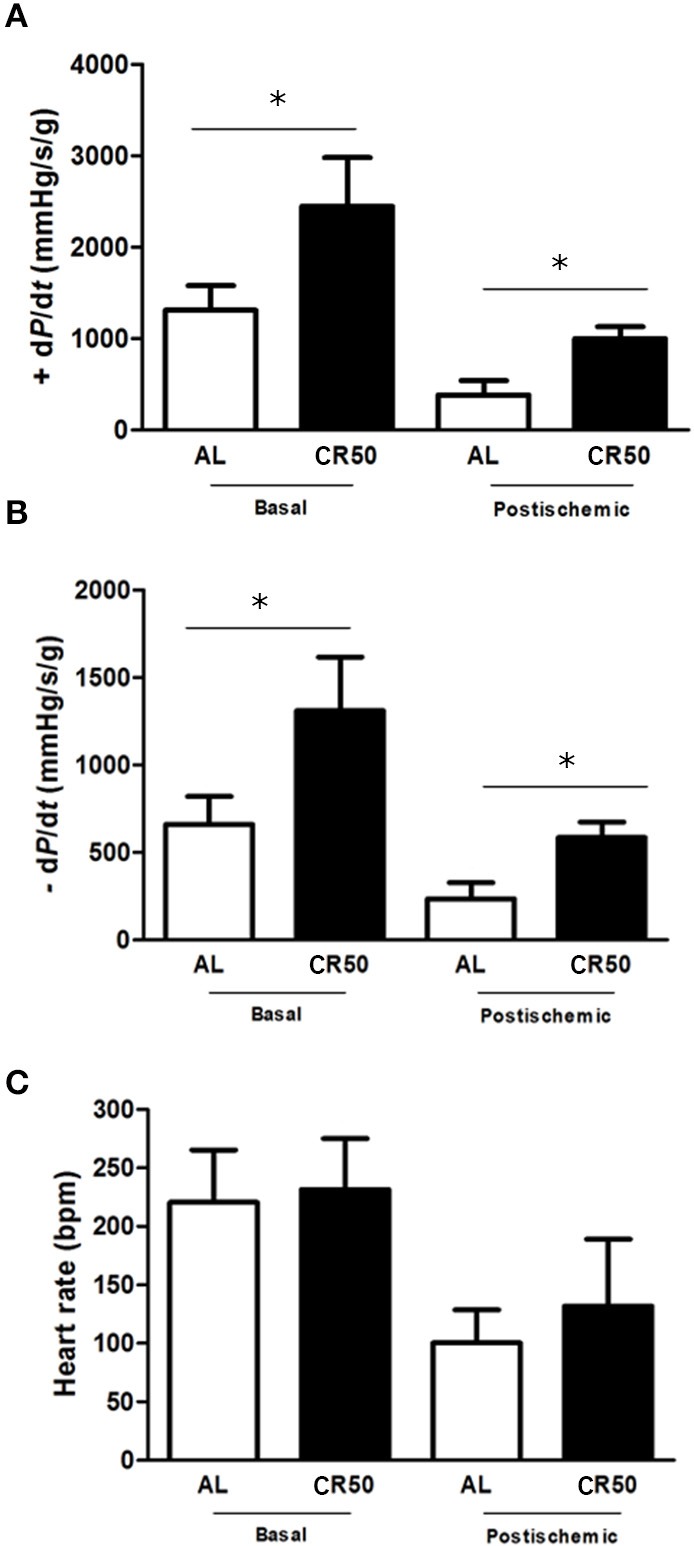
**Severe calorie restriction improves basal and post-ischemic cardiac function**. *Ad Libitum* (AL) and 50% Calorie Restricted (CR50) rats. Contractility index (+dP/dt) **(A)**, relaxation index (−dP/dt) **(B)** and heart rate **(C)** of *Ad Libitum* (AL) and 50% Calorie Restricted (CR50) rats. Data are presented as mean ± SD. ^*^P < 0.05. Anova one-way followed by Tukey *post-hoc* test.

To address if reductions of cardiometabolic risk factors in CR50 rats were followed by an improvement of direct cardiac function, we performed Langendorff analysis (Figure 1). Confirming our previous study Melo et al. (2013), CR50 rats showed increased basal cardiac function as evidenced by increased contractility (+dP/dt) [AL (*n* = 9): 1310 ± 96, CR50 (*n* = 11): 2448 ± 177 mmHg/s-1/g-1, Figure 1A] and relaxation (−dP/dt) [AL (*n* = 9): 660 ± 56, CR50 (*n* = 11): 1310 ± 125 mmHg/s-1/g-1. Figure 1B] indexes without change of intrinsic heart rate [AL (*n* = 9): 220 ± 16, CR50 (*n* = 11): 231 ± 13 bpm, Figure 1C]. In order to investigate if the CR would also have cardioprotective effects in response to a specific stress condition, we subjected the hearts to 20 min of global ischemia followed by an equal period of reperfusion. Compared with AL, CR50 hearts had higher cardiac function at the post-ischemic period as shown by higher +dP/dt [AL (*n* = 8): 384 ± 55, CR50 (*n* = 9): 1001 ± 132 mmHg/s-1/g-1, Figure 1A] and –dP/dt [AL (*n* = 8): 236 ± 34, CR50 (*n* = 9): 585 ± 33 mmHg/s-1/g-1. Figure 1B] with no significant difference in heart rate [AL (*n* = 8): 100 ± 14, CR50 (*n* = 9): 131 ± 57 bpm, Figure 1C]. Of note, despite an expected reduction in cardiac function after global ischemic challenge in both groups, the ± dP/dt indexes in post-ischemic CR50 hearts were similar to that seen in AL hearts at baseline levels.

**Figure 2:**
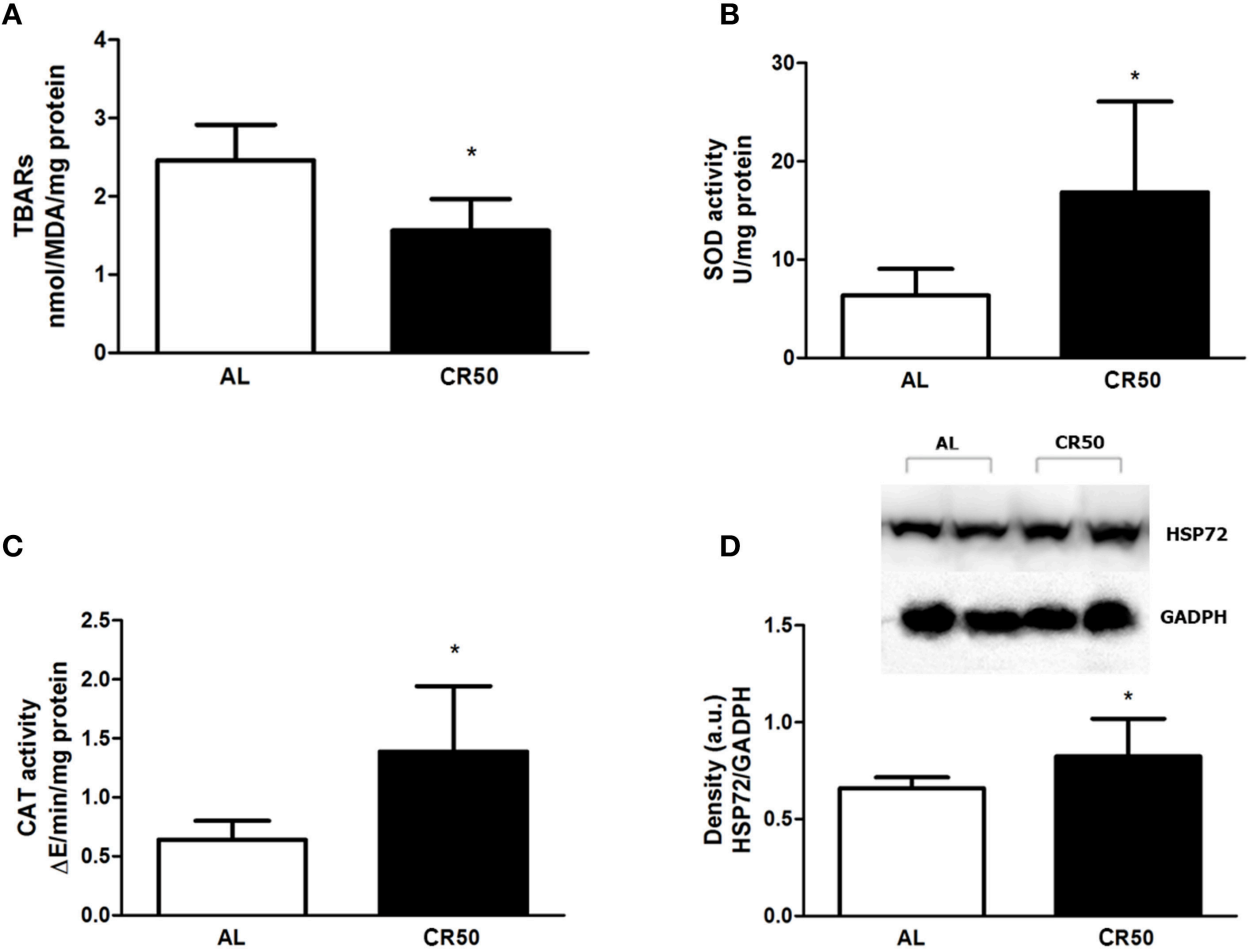
**Severe calorie restriction reduces oxidative stress in post-ischemic hearts**. *Ad Libitum* (AL) and 50% Calorie Restricted (CR50) rats. **(A)** TBARs levels, **(B)** SOD activity, **(C)** CAT activity, and **(D)** expression levels of HSP72 of *Ad Libitum* (AL) and 50% Calorie Restricted (CR50) rats. Data are presented as mean ± SD. ^*^P < 0.05 AL vs. CR50 rats. *T student* test.

In order to further understand the mechanisms involved in the cardioprotective effects of our CR protocol in I/R injury, we assessed cardiac oxidative stress by measuring lipid peroxidation, catalase and superoxide dismutase activities in post-ischemic hearts. As shown in Figure 2, post-ischemic CR50 hearts had lower levels of lipid peroxidation (AL: 2.46 ± 0.14, CR50: 1.56 ± 0.13 nmolMDA/mg protein, Figure 2A), as well as higher activities of SOD [AL (*n* = 8): 6.3 ± 0.9, CR50 (*n* = 9): 16.8 ± 3.4 U/mg protein, Figure 2B], and CAT [AL (*n* = 8): 0.6 ± 0.05, CR50 (*n* = 9): 1.4 ± 0.2 ΔE/min/mg protein, Figure 2C] compared with post-ischemic AL hearts. Additionally, HSP72 content was higher in post-ischemic CR50 hearts (AL: 0.65 ± 0.02, CR50: 0.82 ± 0.09 a.u., Figure 2D).

## Discussion

In the current study, we evaluated the cardioprotective effects of SCR since birth to the age of 3 months in Wistar rats. First, we found that CR50 rats presented a reduction in main cardiometabolic risk factors, as shown by (i) diminished adiposity index and visceral fat weight; (ii) improvement of the plasma lipid profile and atherogenic index; (iii) improvement of the glucose tolerance and insulin response, indicating a higher insulin sensitivity; and (iv) reduced systolic blood pressure, cardiac double product index and basal metabolic rate. Moreover, CR50 rats showed increased cardiac function both at baseline levels (45% > AL rats) and during post-ischemic period (60% > AL rats) which may be explained by a decreased cardiac oxidative stress and increased HSP72 content.

The effects of SCR on cardiac function have been poorly investigated with some controversial results. Although most studies supported the notion that SCR induces positive effects (Broderick et al., 2002; Peron et al., 2005; Han and Ren, 2010; Yamagishi et al., 2010; Melo et al., 2013), some groups demonstrated that SCR has negative effects (Okoshi et al., 2001; Sugizaki et al., 2009; de Tomasi et al., 2009) on the heart. The main explanation for these discrepancies is related to the different experimental conditions. Of note, the period of life in which the CR is initiated is thought to be critical in determining its effects (Han and Ren, 2010). Recently, we found that SCR imposed since birth to the age of 3 months had positive effects on basal cardiac function of Wistar rats (Melo et al., 2013).

Based on our previous data (Melo et al., 2013), two questions were left open regarding the apparent beneficial effects of our CR protocol on heart function. First, we speculated that the increased basal heart function in CR50 rats could be a transitory adaptive change that occurs, for example, in response to cardiometabolic disorders, such as increased blood pressure (Young, 2010). In this study, we discarded this possibility, since the increased basal heart function of CR50 rats was followed by marked positive effects on cardiometabolic parameters. CR50 rats also had a significantly lower basal metabolic rate compared with AL rats. These improvements are in keeping with previous results observed after a traditional moderate long term CR (Park et al., 2005; Niemann et al., 2010) and they have been linked to an adaptative reduction in energy expenditure. The decreased basal metabolic rate has been postulated as one of the mechanisms by which CR exerts cardioprotection, probably through a decrease in ROS generation and a subsequent organ damage reduction (Hulbert, 2010; Kawahara et al., 2014).

The other question left unanswered in our previous study was if, despite the increased basal response, CR50 heart function would be more susceptible to a specific stress. To that end we performed I/R experiments. In fact, CR50 rats not only showed significantly increased heart function at basal levels (confirming our previous findings), but also during the post-ischemic period compared to AL hearts. The post-ischemic function of CR50 hearts actually was similar to the basal levels of AL hearts and 60% higher than the post-ischemic levels of AL hearts. These findings are supported by other studies showing that a long-term SCR protects heart function during the post-ischemic period in adult (Broderick et al., 2002) and senescent (Abete et al., 2002) rats. At baseline levels, Broderick et al. (2002) did not find differences in cardiac function between AL 11-month old rats and age-matched rats restricted by 45% for 32 weeks. However, the post-ischemic cardiac function of CR rats was 28% higher than in AL rats. Abete et al. (2002) also did not observe differences at baseline levels; Yet at the post-ischemic period, 24-month old rats restricted by 40% for 12 months had 40% higher cardiac function than AL age-matched rats. In the present study, SCR rats had not only a higher cardiac function at the post-ischemic period (60% higher than AL rats) but also at baseline levels (45% higher than AL rats). Our results support data obtained from Han and Ren (2010), which suggest that the sooner the CR is started, the greater are its benefits. Thus, we believe that hearts of adult rats, which had started SCR at the first postnatal weeks, may adapt to a condition of reduced energy availability, thereby resulting in an increased cardiac efficiency.

A possible mechanism involved in these benefits, especially the preservation of the cardiac function at the post-ischemic period, may be a lower increase in ROS generation in response to an ischemic insult. Low levels of ROS regulate a variety of key mechanisms involved in the homeostasis of heart function (Hancock et al., 2001; Cadenas, 2004). During I/R injuries ROS is generated at high levels which is associated with cardiac dysfunction (Chen and Zweier, 2014; Silachev et al., 2014). The increase in lipid peroxidation, especially within the sarcoplasmic reticulum, is thought to be a determinant for impaired calcium handling and contractile dysfunction during I/R events (Dhalla et al., 2000; Kaplan et al., 2003). Some studies have proposed that a long-term CR leads to increased tolerance against I/R insult by reducing ROS generation (Shinmura et al., 2011; Walsh et al., 2014) and/or increasing antioxidant activity during the cardiac post-ischemic period (Chandrasekar et al., 2001; Walsh et al., 2014). Therefore, we also attributed the improved cardiac function of CR50 rats at the post-ischemic period to the higher SOD and CAT activities, which might be responsible for the lower levels of lipid peroxidation compared to post-ischemic AL hearts.

The HSP family, especially HSP72, is believed to provide myocardial protection against increased ROS generation during I/R events (Suzuki et al., 2002; Powers et al., 2008). HSP72 is strongly activated during I/R insults and triggers a counter-regulatory action against cellular oxidative damage by modulating the SOD (Suzuki et al., 2002) and CAT (Colotti et al., 2005) activities. To our knowledge, there are no studies evaluating the levels of HSP72 expression in response to I/R injury in SCR rats. Here, we showed that SCR hearts subjected to I/R insult presented higher HSP72 levels which may cause an increase in the antioxidant response preserving cardiac function. However, a complete characterization of the mechanisms underlying these cardioprotective effects was beyond the scope of the present study. Thus, future studies should evaluate if cardioprotective effects of SCR since birth would be observed in other situations. For example, if this protective effect would be lost after a period of refeeding or even if it would be maintained later in life or in pathological conditions.

In conclusion, our findings revealed that SCR from birth to adult age reduces the main cardiometabolic risk factors and directly improves cardiac function at baseline and after an I/R insult. This latter effect may be a result of a decrease in ROS damage through increased antioxidant responses which could be triggered by an increase in HSP72 content.

## Author contributions

Conceived and designed the experiments: DM, MD; performed the experiments: DM, LC, CS, BM, and KC; analyzed the data: DM; contributed reagents/materials/analysis tools: CS, EV, FM, EE; wrote the paper: DM, MD, AF, and SG.

### Conflict of interest statement

The authors declare that the research was conducted in the absence of any commercial or financial relationships that could be construed as a potential conflict of interest.
